# Cytotoxicity of *Cricula triphenestrata* Cocoon Extract on Human Fibroblasts

**DOI:** 10.1155/2012/493075

**Published:** 2012-08-05

**Authors:** Siti Sunarintyas, Widowati Siswomihardjo, Alva Edy Tontowi

**Affiliations:** ^1^Department of Dental Biomaterial, Faculty of Dentistry, Gadjah Mada University, Yogyakarta 55281, Indonesia; ^2^Department of Biomedical Engineering, School of Graduate Studies, Gadjah Mada University, Yogyakarta 55281, Indonesia; ^3^Department of Mechanical and Industrial Engineering, Faculty of Engineering, Gadjah Mada University, Yogyakarta 55281, Indonesia

## Abstract

*Objectives*. The aim of this paper was to evaluate the cytotoxicity of Indonesian silkworm cocoon extract of *Cricula triphenestrata* on human fibroblasts. *Methods and Materials*. The cocoon shells of the silkworm *Cricula triphenestrata* were degumming. The shells were mixed with an aqueous solution of 0.3% Na_2_CO_3_ at 98°C for 1 hour. The solution was then dialyzed in cellulose membranes against deionized water for 3 days. The cocoon shells extract powder was collected via rotary evaporation and dried under freeze dryer. Cell culture medium was exposed to *Cricula triphenestrata* cocoon extract (0.01–100 **μ**g/mL) for 24 hours. The primary human gingival fibroblasts were exposed to the treated cell culture medium for 24 hours. Cytotoxicity evaluation was done by MTT method. The data were analyzed by one-way ANOVA. *Result*. The result revealed no significant cytotoxicity of *Cricula triphenestrata* cocoon extract against human fibroblasts at a concentration up to 100 **μ**g/mL (*P* > 0.05). *Conclusion*. *Cricula triphenestrata* cocoon extract was not cytotoxic on human gingival fibroblast cells.

## 1. Introduction

Many acute and chronic injuries require bone graft substitutes. Current options for bone graft substitutes include autograft, allograft, and synthetic materials. Each of the options has limitations, such as the need for second site of surgery, limited supply, inadequate size and shape, and the morbidity associated with donor site [1]; thus, there remains a need for the new option.

Synthetic hydroxyapatite is widely used as a bone substitute material because it can bond directly to the living bone and has excellent biocompatibility and bioactivity. However, hydroxyapatite does not have enough mechanical properties to act as substitute in loading-bearing parts of the human skeleton. Hydroxyapatite shows a higher Young's modulus and lower toughness than cortical bone [2]. It has been desired to develop a material that has mechanical properties analogous to those of natural bone. It is known that human bone has a three-dimensional woven apatite-polymer structure made of inorganic apatite crystals and organic collagen fibers [3]. Bone consists of an organic-inorganic hybrid with a characteristic structure that leads to specific mechanical properties such as high fracture toughness and flexibility. It is a good strategy to mimic bone structure in the design of bone-repairing materials. From this point of view, the fabrication of hybrid materials consisting of apatite and natural organic polymer can be expected to be a good strategy to obtain bone repairing materials that have both a bone bonding ability and mechanical properties similar to those of natural bone.

Previous study [4] reported that apatite-coated silk scaffolds can combine the osteoconductive properties of bioceramics with the mechanical resilience of polymer. Silk scaffolds combined with apatite promoted cellular attachment and bone formation *in vitro*, providing an appropriate osteogenic environment for tissue engineering. *In vivo* research on mongrel dogs by Zhao et al. [5] showed that apatite-silk scaffolds could be successfully used to repair mandibular critical size border defects. The premineralization of the porous silk protein scaffold provided an increase osteoconductive environment for the cells to regenerate sufficient new bone tissue.

Silks are fibrous proteins with remarkable mechanical properties produced in fiber formed by silkworms and spiders [6]. Previous studies reported silks contained two natural macromolecular proteins namely, sericin and fibroin. Sericin comprised granular and high-molecular, water soluble glycoproteins and it acted as a protein glue to fix fibroin fibers together in the cocoon [7]. In silk textile processing, sericin is usually removed, resulting in fine silk fibers. The resulted fibroin fibers can be used to make fabrics. 

Sericin is useful because of its properties. The protein which resists oxidation is antibacterial, UV-resistant, and absorbs and releases moisture easily. Sericin can be cross-linked, copolymerized, and blended with other macromolecular materials, especially artificial polymers, to produce materials with the improved properties. The protein is also used as an improving reagent or a coating material for natural and artificial fibers, fabrics, and articles. Sericin composites are useful as degradable biomaterials, biomedical materials, polymers for forming articles, functional membranes, fibers, fabrics, and articles [8, 9]. Sericin has a potential to facilitate apatite deposition and can be useful as polymer material in the fabrication of hybrid materials analogous to bone through biomimetic processes [10].

Research regarding the potentials of Indonesian silk is still limited. Indonesia is well known for its silk textile derived from wild silkworm cocoon of *Cricula triphenestrata*. *Cricula triphenestrata *is one of the world wild species of silkworms which only habitats in Java island. *Cricula triphenestrata* produces golden silk floss which is very luxurious and amazing [9]. The use of discarded sericin of *Cricula triphenestrata* cocoon extract from the water waste of silk textile industry as biomaterials will be beneficial for the local silk textile industry and also the development of natural biomaterials as bone substitute.

Biomaterials may have low, medium, or high potential risk to human safety depending on the type and the extent of patient contact. One of the International Standards [11] recommends the appropriate steps for the biological assessment of medical devices *in vitro* assessment of cytotoxicity of new biomaterials. In this primary screening, we aimed to evaluate the cytotoxicity of *Cricula triphenestrata* cocoon extract on human gingival fibroblasts.

## 2. Materials and Method

### 2.1. Materials

**Figure 1:**
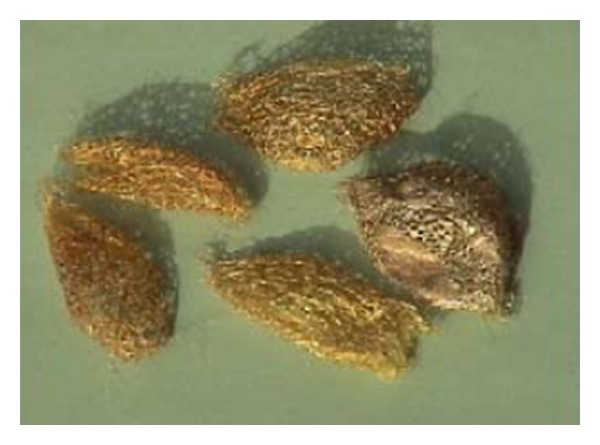
The cocoon shells of the silkworm *Cricula triphenestrata*.

The cocoon shells of the silkworm *Cricula triphenestrata* (Figure 1) were obtained from PT Yarsilk Gora Mahottama Textile Industri at Yogyakarta, Indonesia. The cocoon shells were taken from Karang Tengah Forest at Kabupaten Bantul, Yogyakarta, Indonesia. The medium of RPMI 1640, Dulbecco's Modified Eagle's Minimum Essential medium (DMEM), penicillin, streptomycin, amphotericin, and trypsin were obtained from Gibco (Carlsbad, CA, USA). All other chemicals were analytical or pharmaceutical grade and obtained from Sigma-Aldrich Chemicals (Bornem, Belgium).

### 2.2. Preparation of *Cricula triphenestrata* Cocoon Extract

The cocoon shells of the silkworm *Cricula triphenestrata*, were mixed with an aqueous solution of 0.3% Na_2_CO_3_ (w/v) at 98°C for 1 hour. The solution was then dialyzed in cellulose membranes (MW = 3500 g/mol) against deionized water for 3 days by changing the water daily to remove the ions and other impurities. The cocoon extract powder was collected via rotary evaporation and drying under freeze dryer [12].

### 2.3. Cell Culture

Human gingival fibroblasts were obtained from biopsies of the attached gingival of sound permanent molar teeth of healthy persons. Informed consent based on an appropriate protocol was obtained from the donors. The biopsies were stored at 4°C for at most 24 hours in collection medium (RPMI 1640 supplemented with penicillin 100 U/mL, streptomycin 100 mg/mL, and amphotericin 2.5 mg/mL) prior to amplification. 

The gingival tissues were cut into 1 to 2 mm^3^ pieces then washed three times by RPMI 1640. After that, the cut biopsies were placed into 25 cm^2^ tissue culture flasks. The explants were incubated with culture medium consisting of DMEM 90%, 10 mM HEPES, glucose (4.5 g/L), NaHCO_3_ (3.7 g/L), penicillin (100 U/mL), streptomycin (100 mg/mL), and amphotericin (2.5 mg/mL), supplemented with 10% heat-inactivated fetal calf serum (FCS). The tissue samples were grown at 37°C in a humidified atmosphere of 10% carbon dioxide in the air. When outgrowth of cells was observed, the medium was replaced twice weekly until cells reached confluence. Cells were detached from the monolayer by a brief treatment with trypsin-EDTA (0.25% trypsin, 0.02% EDTA) and recultured in 75 cm^2^ tissue flasks until confluent monolayer was reobtained [13]. 

### 2.4. MTT Cytotoxicity Test

The MTT cytotoxicity test is based on ISO 10993-5 [14] of Biological Evaluation of Medical Devices, Part 5: Tests for *In Vitro* Cytotoxicity. Cells (1 × 10^5^ cells/mL) in DMEM of 50 *μ*L were seeded into 96-well plates and maintained in culture for 24 hours to form a semiconfluent monolayer. They were then exposed to the *Cricula triphenestrata* cocoon extracts (50 *μ*L) over a range of 0.01–100 *μ*g/mL concentration. After 24-hour exposure, the formazan formations were determined for each treatment concentration by ELISA reader at a wavelength of 570 nm. The relative viability of the treated cells as compared to the control cells were expressed as the % cytoviability, using the following formula:

(1) $$\begin{array}{c} \%\text{Cytoviability}=\frac{100\%\times {\text{OD}}_{570\;\;\text{treated}}}{{\text{OD}}_{570\;\;\text{control}}}, \end{array}$$

where OD_570  treated_ is mean value of the measured optical density of the treated cells; OD_570  control_ is mean value of the measured optical density of the control cells.

The data were presented as means ± standard deviation (SD). Statistical analysis was performed using analysis of variance (ANOVA) to determine the effect of cocoon extract concentration on the fibroblast cells cytoviability.

### 2.5. IC_50_ Determination

This study used IC_50_ determination by the standard curve method. A standard curve was performed based on the extract concentrations numbers versus the cytoviability percentage value. The extract concentrations used were 100, 50, 25, 12.5, 6.25, 3.125, 1.563, 0.781, and 0.010 *μ*g/mL. Each concentration was performed in four replicates. The determined cytoviability value was plotted in the standard curve. This research used the cytoviability values that only fall in the linear range of the standard curve to reduce error. A linear regression analysis was performed regarding to the corresponding concentration numbers and cytoviability values. The regression formula was *Y* = *aX* + *b*. The IC_50_ value was estimated using the fitted line of the linear regression as IC_50_ = (0.5 – *b*)/*a*.

## 3. Results

The result of relative viability of the fibroblast treated cells as compared to the control cells was expressed in Table 1. 

Table 1 showed that increased concentration of the *Cricula triphenestrata* cocoon extract exposure on the cells resulted in the decreasing of cytoviability percentage of human gingival fibroblast cells. Further statistical analysis by ANOVA was shown in Table 2, which described that there was not any significant influence of the treated extracts concentration on the cytoviability of human gingival fibroblast (*P* > 0.05). 

The IC_50_ value of *Cricula triphenestrata* cocoon extract for a battery of fibroblast cells was obtained by regression analysis of the corresponding dose-response curve. The regression formula for the dose-response curve was *Y* = 67.630 – 0.390*X*. By the formula, determination of IC_50_ was 172 *μ*g/mL.

## 4. Discussion

In the field of biomaterials, it is necessary to consider aspects of security, such as elimination of cytotoxicity and other harmful effects of the material to be used [15]. By definition, the cytotoxicity of an agent means the toxicological risks caused by a material or its extract in a cell culture [16]. The interactions of the materials and their components with the cells at a molecular level are responsible for tissue reactions, such as inflammation, necrosis [17], immunological alterations, genotoxicity [18], and apoptosis [19].

During the last years, the interest of *in vitro* systems as an alternative to animal experiments in toxicological research has been steadily increasing. Cytotoxicity testing includes numerous methods, both qualitative and quantitative. In this study we used indirect test, in which the rate of cell growth (cell number) and the metabolic activity (MTT) have indicated the degree of cytotoxicity of *Cricula triphenestrata* cocoon extract.

Table 1 shows the effect of *Cricula triphenestrata* cocoon extract on human gingival fibroblast cells viability measured by MTT test. MTT is a yellow water-soluble tetrazolium dye which is reduced by live cells to a purple formazan product insoluble in aqueous solutions. The amount of formazan generated is directly proportional to the number of viable cells [12]. As can be seen from Table 1, the cocoon extract exposure of 0.01–100 *μ*g/mL during 24 hour of incubation induces the cytoviability of the fibroblast to be 71.184–64.486% in comparison to the control. The highest inhibition effect is induced by 100 *μ*g/mL extract concentration and the weakest is on 0.01 *μ*g/mL one. Although further statistical analysis shows no significant difference among the concentrations, but it is proved that there is an increasing cytoviability on the decreasing of the extract concentration exposure. It means that more concentration exposure tends to lower the fibroblast cytoviability.

The half maximal inhibitory concentration (IC_50_) is a measure of the effectiveness of a compound in inhibiting biological or biochemical function. This quantitative measure indicates how much a particular substance is needed to inhibit a given biological process by half. In other words, it is the half maximal (50%) inhibitory concentration (IC) of a substance (50% IC, or IC_50_) [20]. The score of IC_50_ represents the concentration of a drug that is required for 50% inhibition *in vitro *[21]. In this study, the determination of IC_50_ by the formula of *Y* = 67.630 – 0.390*X* is 172 *μ*g/mL. It means that the highest treated concentration of 100 *μ*g/mL cocoon extract reveals no significant cytotoxicity against human fibroblasts (less than 172 *μ*g/mL). 

Previous study reported that the IC_50_ of *Bombyx mori* silkworm cocoon extract on Vero cells was 230 *μ*g/mL [22]. Comparing to the IC_50_ of *Cricula triphenestrata* cocoon extract in this study, the *Cricula triphenestrata* cocoon extract is lower than the *Bombyx mori*. It means that the *Cricula triphenestrata* cocoon extract is more cytotoxic than the *Bombyx mori*. Previous study reported that there was a correlation between environmental condition and the protein production of cocoon [23]. *Cricula triphenestrata* is a wild silkworm found only in Java island forest in Indonesia. It can be found in guava leaf, cashew leaf, and hardwood leaf. *Bombyx mori* silkworms are usually growth by the farmers to produce silk fabrics. They were usually fed with only mulberry leaves. The difference between the environmental condition and food consumption may influence the cocoon characteristic including the cytotoxicity.

In the present study, the cytotoxicity of *Cricula triphenestrata* cocoon extract which is aimed to be used as bone substitute is analyzed using a cell culture model of primary human gingival fibroblasts. Like other tissues, normal fibroblast function is critical to maintain the periodontal tissue function for optimal healing. Gingival fibroblasts are chosen due to their availability and culturing characteristics [13]. 

## 5. Conclusion

Biomaterials may have potential risk to human safety. Accurate biological assessment of the proposed medical biomaterial is needed. Based on the research of the cytotoxicity of Indonesian silkworm cocoon extract of *Cricula triphenestrata* on human fibroblasts, it was concluded that *Cricula triphenestrata* cocoon extract up to 100 *μ*g/mL was not cytotoxic on human gingival fibroblast cells. By this finding, further researches are proposed to develop the *Cricula triphenestrata* cocoon extract as an alternative biomaterial for bone substitute.

## Figures and Tables

**Table 1:** Percentage of cytoviability of human gingival fibroblasts treated by *Cricula triphenestrata* cocoon extract.

Concentration (*μ*g/mL)	% Cytoviability ± SD
100.000	64.486 ± 0.794
50.000	65.995 ± 0.365
25.000	65.998 ± 0.564
12.500	66.298 ± 0.237
6.250	66.698 ± 0.684
3.125	67.947 ± 0.329
1.563	69.433 ± 0.414
0.781	70.259 ± 0.248
0.010	71.184 ± 0.309

**Table 2:** ANOVA summary of the effect of cocoon extracts concentration on fibroblast cells.

	Sum of square	df	Mean square	F	Sig.
Between groups	1174.952	8	146.869	647.248	0.061
Within groups	4.084	18	0.227		

Total	1179.037	26			

## References

[1] LeGeros RZ (2002). Properties of osteoconductive biomaterials: calcium phosphates. *Clinical Orthopaedics and Related Research*.

[2] Hench LL, Wilson J (1993). Introduction. *Introduction to Bioceramics*.

[3] Park JB, Lakes RS (1992). Structure property relationship of biological materials. *Biomaterials: An Introduction*.

[4] Kim HJ, Kim UJ, Kim HS (2008). Bone tissue engineering with premineralized silk scaffolds. *Bone*.

[5] Zhao J, Zhang Z, Wang S (2009). Apatite-coated silk fibroin scaffolds to healing mandibular border defects in canines. *Bone*.

[6] Altman GH, Diaz F, Jakuba C (2003). Silk-based biomaterials. *Biomaterials*.

[7] Wang YZ, Kim HJ, Vunjak-Novakovic G, Kaplan DL (2006). Stem cell-based tissue engineering with silk biomaterials. *Biomaterials*.

[8] Zhang YQ (2002). Applications of natural silk protein sericin in biomaterials. *Biotechnology Advances*.

[9] Wu JH, Wang Z, Xu SY (2007). Preparation and characterization of sericin powder extracted from silk industry wastewater. *Food Chemistry*.

[10] Takeuchi A, Ohtsuki C, Miyazaki T, Tanaka H, Yamazaki M, Tanihara M (2003). Deposition of bone-like apatite on silk fiber in a solution that mimics extracellular fluid. *Journal of Biomedical Materials Research A*.

[11] ISO 10993-1 Biological evaluation of medical devices—part 1.

[12] Zhang F, Zhang Z, Zhu X, Kang ET, Neoh KG (2008). Silk-functionalized titanium surfaces for enhancing osteoblast functions and reducing bacterial adhesion. *Biomaterials*.

[13] Janke V, Neuhoff NV, Schlegelberger B, Leyhausen G, Geurtsen W (2003). TEGDMA causes apoptosis in primary human gingival fibroblasts. *Journal of Dental Research*.

[14] ISO and 10993-5 Biological evaluation of medical devices—Part 5.

[15] Dufrane D, Cornu O, Verraes T (2001). In vitro evaluation of acute cytotoxicity of human chemically treated allografts. *European Cells and Materials*.

[16] Cao T, Saw TY, Heng BC, Liu H, Yap AUJ, Ng ML (2005). Comparison of different test models for the assessment of cytotoxicity of composite resins. *Journal of Applied Toxicology*.

[17] Accorinte MLR, Loguercio AD, Reis A, Muench A, Araújo VC (2005). Adverse effects of human pulps after direct pulp capping with the different components from a total-etch, three-step adhesive system. *Dental Materials*.

[18] Kleinsasser NH, Wallner BC, Harréus UA (2004). Genotoxicity and cytotoxicity of dental materials in human lymphocytes as assessed by the single cell microgel electrophoresis (comet) assay. *Journal of Dentistry*.

[19] Paranjpe A, Bordador LC, Wang MY, Hume WR, Jewett A (2005). Resin monomer 2-hydroxyethyl methacrylate (HEMA) is a potent inducer of apoptotic cell death in human and mouse cells. *Journal of Dental Research*.

[20] Food and Drug Administration (FDA) (1997). Dietary supplements containing ephedrine alkaloids: proposed rule. *Federal Register*.

[21] Cheng YC, Prusoff WH (1973). Relationship between the inhibition constant (K1) and the concentration of inhibitor which causes 50 per cent inhibition (I50) of an enzymatic reaction. *Biochemical Pharmacology*.

[22] Mondal M, Trivedy K, Kumar SN (2007). The silk proteins, serecin and fibroin in silkworm Bombyx mori Linn. *Caspian Journal of Environmental Science*.

[23] Dicko C, Kenney JM, Vollrath F (2006). *β*-silks: enhancing and controlling aggregation. *Advances in Protein Chemistry*.

